# Cultural Asymmetry Between Perceptions of Past and Future Personal Change

**DOI:** 10.3389/fpsyg.2019.00885

**Published:** 2019-04-30

**Authors:** Tieyuan Guo, Roy Spina

**Affiliations:** ^1^ University of Macau, Macau, China; ^2^ University of Chichester, Chichester, United Kingdom

**Keywords:** culture, change, subjective temporal distance, implicit theories, memory

## Abstract

Research has shown that Westerners expect less change to occur in the future than they recall having occurred in the past. The present research investigated how recalled change and anticipated change may vary across cultures. Because Chinese perceive past times as being closer to the present than do Westerners, and people believe things tend to change more over a long period of time than over a short period of time, Chinese may perceive smaller changes from the past to the present than do Westerners. Consequently, the asymmetry between recalled change and anticipated change would disappear for Chinese. Four empirical studies revealed that for British participants, recalled changes in the past for personality, values, and the person as a whole were greater than anticipated changes in the future, whereas for Chinese, recalled changes in the past were similar in magnitude as anticipated changes in the future. Studies 2b and 3 further revealed that subjective temporal distance accounted for the cross-cultural differences in the asymmetry between recalled and anticipated changes.

“The past is never where you think you left it.”

—Katherine Anne Porter

## Introduction

“Where do you see yourself five years from now?” A candidate feels anxious when she encounters this frequently asked question in a job interview. Although she believed that she had many improvements in her working skills in the past 5 years, she may have doubts about her further improvements in the next 5 years. Although people generally rely on their memorized past experiences when anticipating future developments, they tend to perceive a greater change in the past than in the future (Gomez et al., 2013; Quoidbach et al., 2013). Would culture play a role in perceived personal change in the past and anticipated change in the future? This paper examines how one’s cultural background may impact the magnitudes of recalled and anticipated personal change.

### Recalled Change and Anticipated Change

People rely on autobiographical memories to construe what happened to them in the past. Autobiographical memory, as well as other types of memory, is not only a reproductive process which allows people to contemplate what actually happened in the past, but also a reconstructive process in which what happened in the past is inferred from the cues available at the present (Bartlett and Bartlett, 1995; Conway and Pleydell-Pearce, 2000). Consequently, the implicit theories people hold can bias their autobiographical memories (Ross, 1989; Wilson and Ross, 2003). People who hold an implicit theory of stability exaggerate the similarity between the present and the past, and those whose implicit theory leads them to believe that change should happen exaggerate the discrepancy between the present and the past. For example, because students generally believed that a skill training program would improve their skills, they recalled their skills before entering the training program as being worse than they had initially reported, thus exaggerating the difference between their present and past skills (Conway and Ross, 1984).

In addition to implicit theories, self-enhancement motivations can also alter people’s memory about themselves in the past (Wilson and Ross, 2001, 2003). People in Western cultures are generally motivated to view their present self positively (Sedikides et al., 2003; Falk and Heine, 2014). One way to achieve and maintain positive views on the current self is to disparage the past self so that people can create an illusion that they had improved from the past. For example, Wilson and Ross (2001) found that college students criticized their past selves and rated their past selves as inferior to their present selves, even though no actual improvement had taken place. In a similar vein, research on assimilation and contrast effects has shown that recalling personal negative events from the distant past can make people feel happier and more satisfied with their current life (Strack et al., 1985).

People not only rely on implicit theories to reconstruct what happened in the past, but also to infer what will happen in the future (Dweck and Leggett, 1988; Chiu et al., 1997; Ji et al., 2001). For example, people who hold an implicit linear theory of change expect that things would be stable in the future or would change in a linear way by continuing the trends in the past. In contrast, people who hold an implicit nonlinear theory of change expect that things change constantly and the change in the future is likely to be circular, i.e., reversing the trend (Ji et al., 2001). Similarly, people who hold entity theories believe that their personalities, values, abilities, etc. are fixed. In contrast, incremental theorists believed that these attributes are malleable (Dweck and Leggett, 1988). Consequently, entity theorists expect that their future selves would be similar to the present ones, whereas incremental theorists expect that their future selves would be different from the present ones. Burnette (2010) found that after diet setbacks in their present weight-loss program, people were more likely to expect success in the future when they believed that their body weight was malleable compared with when they believed that it was fixed.

Interestingly, the magnitude of recalled change in the past and that of anticipated change in the future tend to be different, at least for Westerners (Gomez et al., 2013; Quoidbach et al., 2013). Westerners anticipated that their personal change over a period of time in the future would be smaller in magnitude than the change they recalled experiencing over the same length of time in the past. In one study, Western participants of different ages completed a big five personality inventory, and then they completed the same inventory either as they would have completed it 10 years earlier, or as they would complete it in 10 years. The recalled (and anticipated) personality changes were derived from the discrepancies between the present personality and the recalled personality 10 years ago (and the anticipated personality in 10 years). They found that anticipated personality changes in the future by participants at each age group were smaller in magnitude than the recalled personality changes in the past by another group of participants who were 10 years older. To our interest, the anticipated personality changes in the future were also smaller in magnitude than the recalled changes for participants of the same age, which is of particular interest in the current paper. Similarly, Gomez et al. investigated the perception of past, present, and future life satisfaction of Swiss people at different ages. Again, to our interest, the correlations between the present and the recalled past life satisfactions for young and middle-aged adults were generally smaller than the correlations between their present and anticipated future life satisfactions, indicating that they anticipated less change in life satisfaction in the future than they recalled such change in the past.

In the literature, the association between time perception and recalled/anticipated change was rarely studied. Because any change needs time to take place, we believe people would generally hold the implicit theory that things would change more over a long period of time than over a short period of time, regardless of whether this change is in the past, at the present, or in the future. Thus, time perception may bias our perceptions of change.

### Culture and Time Perception

Culture shapes the way people process time information. Compared to Western cultures, Chinese culture is believed to be primarily past oriented (Kluckhohn and Strodtbeck, 1961; Spears et al., 2000; Brislin and Kim, 2003; Ji et al., 2009; Guo et al., 2012). Compared to Westerners, Chinese considered information pertaining to the past as being more relevant to solving present problems (Ji et al., 2009), emphasized more on traditions and being historical in TV commercials (Cheng and Schweitzer, 1996), and valued past events relatively more than future ones (Guo et al., 2012). For example, although Americans tend to value future events more than similar past ones (Caruso et al., 2008), Chinese place more value on past events than on future ones (Guo et al., 2012).

The perceived temporal distance of the past also varies across cultures, which is most relevant to the present paper. Because of their strong past orientations, Chinese perceive past events and times as being closer to the present than do Westerners (Ji et al., 2009). In one study, compared with Canadians, Chinese reported that the current month 1 year ago was perceived as being closer to the present. Similarly, in another study, final exams from the previous semester were perceived as being closer to the present by Chinese students than by Canadian students, even though the actual temporal distance of the exams was longer for Chinese than for Canadians.

### The Present Research

The current research investigated how culture might influence the asymmetry between recalled personal change in the past and anticipated change in the future. Change and time are closely associated. Any change in the world needs time to take place. Greater time is associated with greater change. Thus, people would hold an implicit theory that things are likely to change more over a long period of time than over a short period of time. Such implicit theory might be applied when people estimate the amount of change across a period of time. That is, they may use perceived temporal distance as a cue to infer the amount of change that happened in the past or would happen in the future. For example, a person who feels that 5 years ago (in the future) is very far away would perceive that he or she has changed (would change) more over the 5 years than another person who feels that 5 years ago (in the future) is very close.

As reviewed earlier, Chinese and Westerners differ in their time perception. Chinese perceive past events and times as being closer to the present than do Westerners (Ji et al., 2009). Therefore, we predicted that although the recalled personal change in the past would be greater than the anticipated change in the future for Westerners, the asymmetry would not exist for Chinese. We further predicted that this cultural variation would be accounted for by perceived temporal distance. We conducted four studies to test these hypotheses. Specifically, in the first two studies, Chinese and British participants reported their present personality (Study 1) and values (Study 2a), as well as their personality and values either 10 years ago, or in 10 years. We then examined whether recalled changes in personalities and values in the past were greater than anticipated changes in the future for Westerners, and whether the asymmetry disappeared for Chinese. Then, in order to explore the underlying mechanism for such cultural variation, we included a subjective temporal distance measure and a self-enhancement measure in Study 2b. In Study 3, we examined the recalled (and anticipated) changes over 1 year, a relatively short period of time, for which participants would have more information in their memory. In addition, we also examined whether participants actually held the belief that a larger period of time is associated with greater change. In these studies, we reported all measures and manipulations.

## Study 1

In this study, British and Chinese participants reported their past, present, and future personalities.

### Methods

#### Participants

Because no existing research allowed us to estimate the effect size, we estimated the number of participants based on the rule of thumb. In addition, because we predicted that Chinese did not differ in their recalled and anticipated personal change, we tried to get more Chinese participants than British participants so that we can have a greater power to detect any potential differences in the recalled and anticipated personal change for Chinese. Specifically, we decided to stop data collection when we got 100 British participants and 150 Chinese participants or when the subject pool closed at the end of the semester when data collection took place, whichever occurred earlier. Sample size was determined before any data analysis. Same rules applied to Studies 2a and 2b. Consequently, 99 undergraduate students (31 males) from a University in Macau, China, and 76 undergraduate students (22 males) from a University in the United Kingdom, participated in the study. Chinese participants (*M* = 18.85, *SD* = 1.24) were younger than British participants (*M* = 20.34, *SD* = 4.09), *p* = 0.001. For all the studies reported in the paper, age had no significant effect when it was included as a covariate in the data analysis, and the inclusion of age in the analysis did not change the pattern of the results. Thus, age will not be further discussed.

#### Procedure

Participants first completed the present personality measure, followed by the past or the future personality measure. Across all the studies reported in the paper, we reported all measures, manipulations, and exclusions.

##### The Present Personality Measure

The Ten-Item Personality Inventory (TIPI) (Gosling et al., 2003) was adopted to measure the present personalities. The TIPI is a short measure of the big five personality dimensions. The big five personality traits have been demonstrated to be culturally universal, i.e., shared by people from both cultures (McCrae and Costa, 1997).

Specifically, participants rated how much they agreed or disagreed that each of the 10 personality items on the scale described them (ranging from 1 = *Disagree strongly* to 7 = *Agree strongly*). Sample items include “*extraverted, enthusiastic*,” and “*conventional, uncreative*.”

##### The Past and Future Personality Measures

After completing the TIPI for the present, participants were randomly assigned to the past or the future condition. In the past condition, they imagined that they had been asked to complete the TIPI 10 years earlier. They rated the TIPI as if they would have done it 10 years before. Similarly, participants in the future condition imagined that they would be asked to complete the TIPI in 10 years. They rated the TIPI as they would do in 10 years. This measure was adapted from Quoidbach et al. (2013).

The questionnaire was first created in English. It was then translated into Chinese by two independent bilinguals. The two translations were compared and all the discrepancies in the translations were discussed until final agreements were reached. The translation was then verified by the first author, who is a Chinese-English bilingual with Chinese as his first language. Chinese participants completed the study in Chinese and British participants completed the study in English. The same translation procedure was applied to all the studies in this paper.

### Results

**Figure 1:**
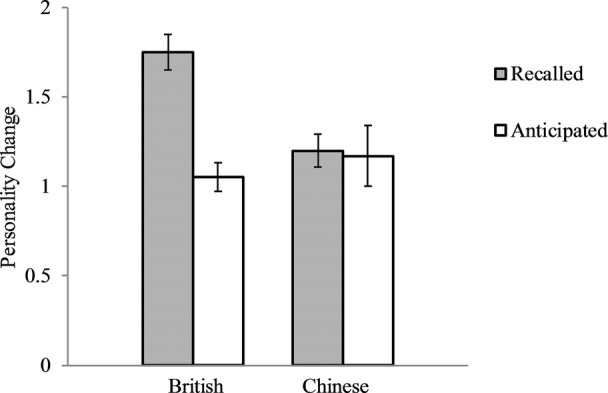
British and Chinese recalled personality changes in the past 10 years and their anticipated personality changes in the future 10 years. Error bars represent standard errors.

For each of the 10 TIPI items, we calculated the absolute value of the difference between participants’ ratings on their present personalities and the ratings on their past or future personalities. We then averaged the absolute changes in the 10 personality items to create the indices for the recalled personality change in the past and the anticipated personality change in the future. See Figure 1 for the results. A 2 (Culture: British vs. Chinese) X 2 (Time: Past vs. Future) ANOVA with the magnitude of change as the dependent variable revealed that the culture by time interaction was significant, *F*(1, 171) = 7.56, *p* = 0.01, partial *ɳ*^2^ = 0.04. Simple effect analysis revealed that, as expected, the magnitude of anticipated personality change in the future (*M* = 1.05, *SD* = 0.51) was smaller than that of recalled personality change in the past (*M* = 1.75, *SD* = 0.63) for British, *F*(1, 74) = 28.54, *p* < 0.001, partial *ɳ*^2^ = 0.28, *Cohen’s d* = 1.22. The analysis on British participants had 80% power to detect an effect size of *Cohen’s d* = 0.65. In contrast, for Chinese, the magnitudes of recalled (*M* = 1.20, *SD* = 0.60) and anticipated (*M* = 1.17, *SD* = 1.19) personality changes did not differ, *p* = 0.89. The analysis had 80% power to detect an effect size of *Cohen’s d* = 0.57. Thus, the results supported the prediction that the magnitude of the recalled change would be greater than that of the anticipated change for British, whereas the asymmetry would not exist for Chinese.

## Study 2a

In Study 2a, we examined recalled and anticipated changes in basic values. In addition, in order to examine if British and Chinese differ in their explicit beliefs regarding changes in the past and in the future, we asked participants to directly report remembered changes in the past and anticipated changes in the future.

### Methods

#### Participants

One hundred and thirty-five Chinese undergraduate students (64 males and 71 females) from a University in Macau, China, and 82 British undergraduate students (18 males and 64 females) from a University in United Kingdom, completed the questionnaire package in the study. Chinese participants (*M* = 18.95, *SD* = 1.11) were younger than British participants (*M* = 22.28, *SD* = 7.01), *p* < 0.001.

#### Procedure

We created online questionnaires with Qualtrics. Participants completed the online questionnaires in research labs. They completed the present and the past or the future basic value measures. Then, they directly reported value changes in the past and in the future.

##### The Present and the Past or the Future Values Measure

Ten core values were taken from the Schwartz Value Inventory (Schwartz, 1992). The 10 core values are basic values shared by people from different cultures (Schwartz, 1992, 2012). Sample items include “power” and “achievement.” Participants were randomly assigned to the past or the future condition. In the past condition, participants rated how important each of the 10 values was (and had been) for them “*today*” (and “*10 years ago*”) on a scale ranging from −1 (*Opposed to my values*), to 0 (*Not important*), to 7 (*Of supreme importance*). Participants in the future condition rated how important these values were for them today and would be for them in 10 years using the same scale.

##### Directly Reported Value Changes in the Past and in the Future

Participants in the past (future) condition reported how much they thought their values had changed (would change) in general over the last (next) 10 years on a scale ranging from 1 (*Not at all*) to 7 (*Very much*).

### Results

**Figure 2:**
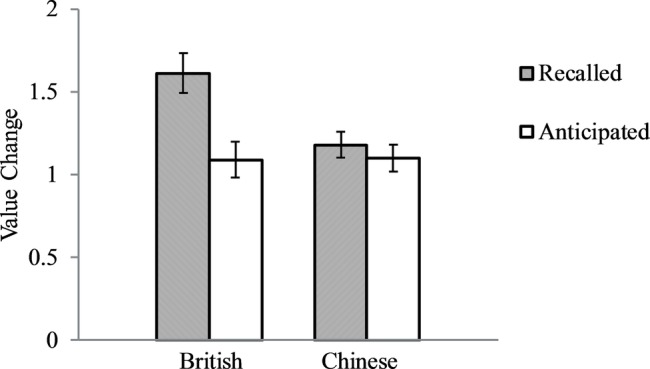
British and Chinese recalled value changes in the past 10 years and their anticipated value changes in the future 10 years. Error bars represent standard errors.

As in Study 1, we calculated the absolute changes in values in the last 10 years and in the next 10 years (Figure 2). One British participant and one Chinese participant did not report their future values, and their absolute changes in values in the future were entered as missing values. Similar analyses as in Study 1 revealed that the culture by time interaction was significant, *F*(1, 211) = 5.43, *p* = 0.021, partial *ɳ*^2^ = 0.025. As expected, for British, the change in values in the past (*M* = 1.61, *SD* = 0.76) was greater than the anticipated change in the future (*M* = 1.09, *SD* = 0.68), *F*(1, 79) = 10.63, *p* = 0.002, partial *ɳ*^2^ = 0.119, *Cohen’s d* = 0.724, whereas for Chinese, the recalled change (*M* = 1.18, *SD* = 0.69) and anticipated change did not differ (*M* = 1.10, *SD* = 0.63), *p* = 0.53.

**Figure 3:**
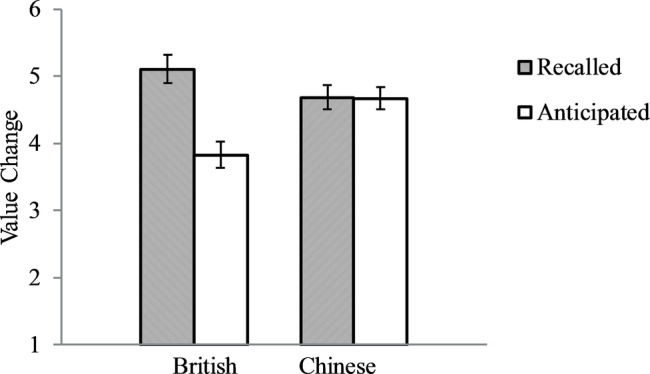
British and Chinese directly reported value changes in the past 10 years and in the future 10 years. Error bars represent standard errors.

Similar findings were obtained for directly reported changes in the past and in the future (Figure 3). The culture by time interaction was significant, *F*(1, 213) = 10.86, *p* = 0.001, partial *ɳ*^2^ = 0.049. British participants reported that their values had changed more over the last 10 years (*M* = 5.10, *SD* = 1.32) than they would change in the next 10 years (*M* = 3.83, *SD* = 1.28), *F*(1, 80) = 19.48, *p* < 0.001, partial *ɳ*^2^ = 0.196, *Cohen’s d* = 0.975, whereas Chinese participants reported that their value change over the past 10 years (*M* = 4.68, *SD* = 1.46) was similar to the change in the next 10 years (*M* = 4.67, *SD* = 1.32), *p* = 0.95. The analyses on British participants had 80% power to detect an effect size of *Cohen’s d* = 0.63. And the analyses on Chinese participants had 80% power to detect an effect size of *Cohen’s d* = 0.49.

Therefore, the cross-cultural asymmetry in recalled and anticipated changes was not only limited to personality as in Study 1. We conceptually replicated the findings with value changes.

## Study 2b

In Study 2b, we further examined general personal change. Besides, we examined the underlying mechanism for the cultural variation in recalled and anticipated changes, namely subjective temporal distance. In addition, self-enhancement motivation could account for the expected discrepancies in anticipated and recalled personal change, at least for British, i.e., British may be motivated to believe that they have improved greatly from the past and their current self is the best, which leaves little room for further improvement in the future (Quoidbach et al., 2013). Therefore, we tested this alternative mechanism by including a measure of self-enhancement.

### Methods

#### Participants

The same participants from Study 2a participated in Study 2b. Participants engaged in irrelevant tasks for about 30 minutes in between the two studies.

#### Procedure

##### Personal Change

Participants completed the questionnaire online. Independent to the random assignment in Study 2a, they were again randomly assigned to the past or the future conditions. In the past (future) condition, participants reported how much they thought they had changed (would change) over the last (next) 10 years on a scale ranging from 1 (*Not at all*) to 7 (*Very much*).

##### Subjective Temporal Distance

Participants reported the subjective temporal distance of their past (future) self. Specifically, participants in the past (future) condition read that “*the past (future) self may feel quite near or far away, regardless of how much time the past (future) self is away from the present. Think about yourself one year ago (one year from now). How far away do you feel yourself one year ago (one year from now) is to the present?*” They rated the subjective temporal distance of their past/future self on a scale ranging from 1 (*Feels like yesterday*/*Feels like tomorrow*) to 7 (*Feels very far away*). Similar subjective temporal distance measures have been used by other researchers (Wilson and Ross, 2001; Ji et al., 2009; Caruso et al., 2013).

##### Self-Enhancement

The self-enhancement measure was taken from Sedikides et al. (2003). Specifically, participants first imagined themselves being a member of a 16-person group. They all worked together to solve business problems. They had a series of business considerations for which they and other group members need to strategize. They imagined exchanging ideas with other group members. After that, they were presented with a list of 16 behaviors, half of them were individualistic (e.g., *engage in open conflict with your group*) and the other half were collectivistic (e.g., *follow the rules according to which your group operates*). Then, they rated how likely they would perform each behavior, compared to their typical group member on an 11-point scale ranging from −5 (*much less likely than the typical group member*) to 5 (*much more likely than the typical group member*) with a middle point 0 (*about the same as the typical group member*). Higher scores indicate greater self-enhancement.

### Results

**Figure 4:**
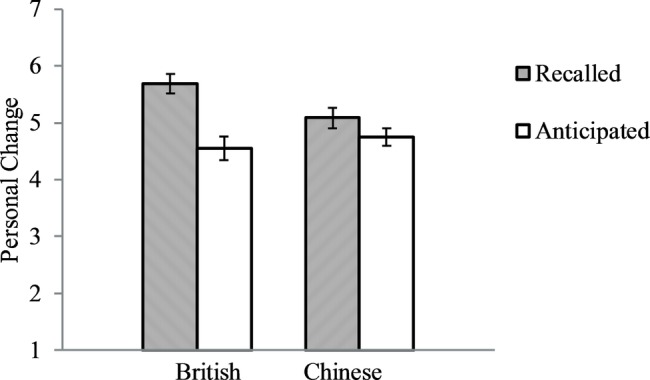
British and Chinese directly reported personal changes in the past 10 years and in the future 10 years. Error bars represent standard errors.

Similar analyses as in the foregoing studies revealed that the culture by time interaction was significant, *F*(1, 213) = 5.56, *p* = 0.019, partial *ɳ*^2^ = 0.025. As expected, British reported that they had changed more in the last 10 years (*M* = 5.69, *SD* = 1.07) than they would change in the next 10 years (*M* = 4.55, *SD* = 1.36), *F*(1, 80) = 17.93, *p* < 0.001, partial *ɳ*^2^ = 0.183, *Cohen’s d* = 0.933. In contrast, Chinese reported that they had changed the same amount in the past 10 years (*M* = 5.09, *SD* = 1.22) as they would change in the next 10 years (*M* = 4.75, *SD* = 1.21), *p* = 0.109. See Figure 4. Thus, the data supported our prediction. As in Study 2a, the analysis on British participants had 80% power to detect an effect size of *Cohen’s d* = 0.63. And the analysis on Chinese participants had 80% power to detect an effect size of *Cohen’s d* = 0.49.

Similar analyses were conducted for subjective temporal distance. The culture by time interaction was significant, *F*(1, 213) = 5.32, *p* = 0.022, partial *ɳ*^2^ = 0.024. The subjective temporal distance for the future self was similar for British (*M* = 4.98, *SD* = 1.66) and for Chinese (*M* = 4.67, *SD* = 1.54), *p* = 0.33. However, as expected, British (*M* = 5.60, *SD* = 1.38) perceived their past selves 10 years ago as being further away from the present than did Chinese (*M* = 4.30, *SD* = 1.51), *F*(1, 106) = 20.10, *p* < 0.001, partial *ɳ*^2^ = 0.159, *Cohen’s d* = 0.894. Thus, we conceptually replicated the findings by Ji et al. (2009) that Westerners perceived past events and times as being further away than did Chinese. Interestingly, further analysis revealed that British participants perceived their past selves as being further away than their future selves at a marginally significant level, *F*(1, 80) = 3.41, *p* = 0.069, *Cohen’s d* = 0.407. In contrast, Chinese perceived the past selves and future selves as being equally distant from the present, *p* = 0.17.

The individualistic and collectivistic self-enhancements had acceptable internal reliabilities, 0.81 and 0.69, respectively. British participants (*M* = −0.08, *SD* = 1.55) had greater self-enhancement in individualistic behaviors than did Chinese participants (*M* = −1.10, *SD* = 1.50), *F*(1, 215) = 22.83, *p* < 0.001, partial *ɳ*^2^ = 0.096, whereas Chinese (*M* = 0.70, *SD* = 1.27) had greater self-enhancement in collectivistic behaviors than did British (*M* = 0.07, *SD* = 1.12), *F*(1, 215) = 13.65, *p* < 0.001, partial *ɳ*^2^ = 0.060.

#### Moderated Mediation Analysis

**Figure 5:**
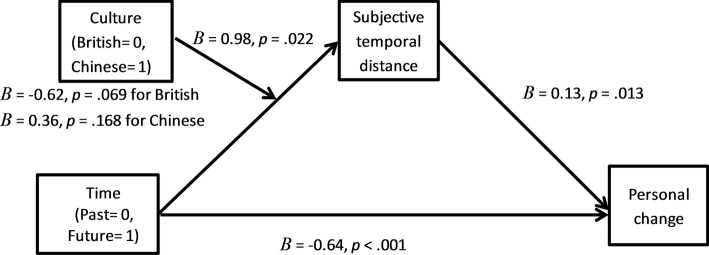
Culture moderated the effect of time condition on subjective temporal distance, which positively predicted personal change.

Following the procedure outlined by Preacher and Hayes (2004), a bootstrapping (*N* = 5,000) with time condition (past = 0 and future = 1) as the predictor, subjective temporal distance as the mediator, culture (British = 0 and Chinese = 1) as the moderator on the effect from culture to subjective temporal distance, and personal change as the dependent variable was conducted (see Figure 5). As expected, the culture by time interaction on subjective temporal distance was significant, *B* = 0.98, *t* = 2.31, *p* = 0.022, and the 95% CI was (0.14, 1.83). The effect of subjective temporal distance on personal change was significant, *B* = 0.13, *t* = 2.52, *p* = 0.013, and the 95% CI was (0.03, 0.23). The further away participants felt their past and future selves were from the present, the more personal changes they recalled or anticipated. The direct effect of time condition on personal change was also significant, *B* = −0.64, *t* = −3.89, *p* < 0.001, and the 95% CI was (−0.97, −0.32). The past selves were perceived as being further away from the present than the future ones. The conditional indirect effect of time condition on personal change was significant for British participants, *B* = −0.08, and the 95% CI was (−0.2493, −0.0006), and nonsignificant for Chinese participants, *B* = 0.05, and the 95% CI was (−0.01, 0.17). The recalled personal changes in the past were greater in magnitude than the anticipated personal changes in the future for British participants, which was partially accounted for by the fact that they perceived their past selves as being further away from the present than their future selves. In contrast, Chinese participants perceived the past and future selves as being equally far away from the present, and for them, the recalled personal change was similar in magnitude as the anticipated personal change. Similar results were obtained with a similar analysis where culture was entered as the predictor, and time condition was entered as the moderator. In short, subjective temporal distance explained the cultural asymmetry between recalled and anticipated personal changes.

In order to test the alternative mediating mechanism of self-enhancement motivations, a bootstrapping analysis with culture as the predictor, individualistic and collectivistic self-enhancements as the mediators, personal change as the outcome, and time condition as the moderator moderating the influence of the mediators on personal change, was conducted. The results showed that although culture had significant influences on both individualistic and collectivistic self-enhancements, *ps* < 0.001, the time condition by individualistic self-enhancement as well as the time condition by collectivistic self-enhancement were not significant, *ps* > 0.47. Thus, the results showed that self-enhancement did not account for the cultural asymmetry between recalled and anticipated personal change.

## Study 3

In Studies 1, 2a, and 2b, we examined changes in a 10-year time frame. Given that the participants were young adults, they might not have had much memory about themselves 10 years ago and thus had to rely on implicit theories to infer the amount of change in the past conditions. In Study 3, in order to examine if the cultural asymmetry holds true for a relatively short time period, i.e., when people have more information about the past in their memory, we examined the recalled (and anticipated) changes over 1 year. In addition, we examined whether people actually held the belief that things tend to change more over a long period of time than over a short period of time.

### Methods

#### Participant

Based on the average effect size (average *Cohen’s d* = 0.963) obtained in Studies 1, 2a, and 2b, we calculated the required sample size that allowed us to have 90% power to find the asymmetry in recalled change in the past and anticipated change in the future for British at the *p* level of 0.05. The results showed that we need 48 British participants. Again, we decided to get a slightly higher number of Chinese participants because we expected the recalled change in the past and anticipated change in the future would be similar in magnitude for Chinese. Consequently, 48 British undergraduate students (11 males and 37 females) from a University in the United Kingdom and 66 Chinese undergraduate students (24 males and 39 females; 3 participants did not report their gender information) from a University in Macau, China, participated in the study. Chinese participants (*M* = 19.70, *SD* = 1.61) were younger than British participants (*M* = 22.31, *SD* = 6.82), *p* = 0.01. Data from one British participant were removed from the analyses because the participant was an Asian.

#### Procedure

We first measured participants’ belief regarding the association between magnitude of change and length of time. Specifically, participants reported how much they agree that “*things tend to change more over a long period of time than over a short period of time*” (long time associated with more change), “*things tend to change less over a long period of time than over a short period of time*” (long time associated with less change), and “*things tend to change the same amount over a long period time as over a short period of time*” (time not associated with change) on scales ranging from 1 (*Strongly disagr*ee) to 7 (*Strongly agree*).

Then, they completed the same questionnaire as in Study 2b, except that the time period was set to 1 year and that the self-enhancement measure was excluded. That is, participants reported recalled personal changes in the last year and anticipated changes in the next year. They also reported the subjective temporal distance of their selves 1 year ago or 1 year in the future.

### Results

#### Beliefs Regarding the Association Between Magnitude of Change and Length of Time

Chinese participants agreed more with the long-time-associated-with-more-change statement (*M* = 5.36, *SD* = 1.01) than with the long-time-associated-with-less-change statement (*M* = 3.33, *SD* = 1.36), and with the time-not-associated-with-change statement (*M* = 3.73, *SD* = 1.18), *ps* < 0.001. Similarly, British participants also agreed more with the long-time-associated-with-more-change statement (*M* = 5.55, *SD* = 1.06) than with the long-time-associated-with-less-change statement (*M* = 2.91, *SD* = 1.33), and with the time-not-associated-with-change statement (*M* = 3.23, *SD* = 1.24), *ps* < 0.001. Moreover, both Chinese and British participants’ ratings on the long-time-associated-with-more-change statement were higher than the middle point of the scale, i.e., *neither agree nor disagree*, *ps* < 0.001. Their ratings on the long-time-associated-with-less-change statement were lower than the middle point of the scale, *ps* < 0.001. In addition, their ratings on the time-not-associated-with-change statement were also lower than the middle point of the scale, *p* < 0.001 for British, and *p* = 0.066 for Chinese. The analyses had 80% power to detect an effect size of *Cohen’s d* = 0.54 for each comparison. Taken together, the findings revealed that both British and Chinese participants hold the belief that things tend to change more over a long period of time than over a short period of time.

#### Personal Change

**Figure 6:**
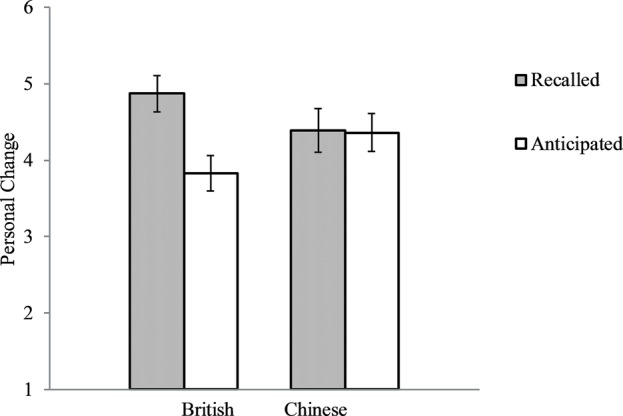
British and Chinese directly reported personal changes in the last year and in the next year. Error bars represent standard errors.

Similar as in foregoing studies, the culture by time interaction was marginally significant, *F*(1, 109) = 3.61, *p* = 0.06, partial *ɳ*^2^ = 0.03. As expected, British reported that they had changed more in the last year (*M* = 4.87, *SD* = 1.14) than they would change in the next year (*M* = 3.83, *SD* = 1.13), *F*(1, 45) = 9.79, *p* = 0.003, partial *ɳ*^2^ = 0.18, Cohen’s d = 0.91. In contrast, Chinese reported that they had changed the same amount in the last year (*M* = 4.39, *SD* = 1.64) as they would change in the next year (*M* = 4.36, *SD* = 1.43), *p* = 0.94. See Figure 6. Thus, the data supported our prediction. The analysis on British participants had 80% power to detect an effect size of *Cohen’s d* = 0.81. And the analysis on Chinese participants had 80% power to detect an effect size of *Cohen’s d* = 0.70.

Similarly, for the subjective temporal distance, the culture by time interaction was marginally significant, *F*(1, 109) = 3.31, *p* = 0.07, partial *ɳ*^2^ = 0.03. The subjective temporal distance for the future self was similar for British (*M* = 3.88, *SD* = 1.62) and for Chinese (*M* = 3.82, *SD* = 1.40), *p* = 0.89. However, as expected, British (*M* = 4.52, *SD* = 1.53) perceived the past self as being further away from the present than did Chinese (*M* = 3.39, *SD* = 1.62), *F*(1, 54) = 6.86, *p* = 0.01, partial *ɳ*^2^ = 0.11. Within-cultural analysis revealed that there was a trend for British participants to perceive the past selves as being further away than the future ones, but it was not significant, *p* = 0.168, *Cohen’s d* = 0.410. The insignificant trend might be a result of relatively small number of participants in the study. A power analysis based on the effect size obtained in Study 2b revealed that in order to allow us to have 90% of power to find the asymmetry in subjective temporal distances at the *p* level of 0.05, we would need 210 British participants, which was much greater than the number of British participants in the current study. Chinese participants perceived the past and the future selves as equally far away from the present, *p* = 0.259.

#### Moderated Mediation Analysis

A similar bootstrapping analysis as in Study 2b with time condition as the predictor, subjective temporal distance as the mediator, culture as the moderator, and change as the outcome was conducted. As expected, the culture by time interaction on subjective temporal distance was significant, *B* = 1.07, *t* = 1.82, *p* = 0.072, the 95% CI was (−0.10, 2.24). The effect of subjective temporal distance on personal change was marginally significant, *B* = 0.19, *t* = 2.28, *p* = 0.02, the 95% CI was (0.02, 0.35). The further away participants felt their past and future selves were from the present, the more personal changes they recalled or anticipated. However, the conditional indirect effects of time condition on personal change were not significant for both British participants, *B* = −0.12, and the 95% CI was (−0.48, 0.02), and Chinese participants, *B* = 0.08, and the 95% CI was (−0.03, 0.35). Again, the nonsignificant indirect effect for British might be a result of the small number of British participants in the study.

**Figure 7:**
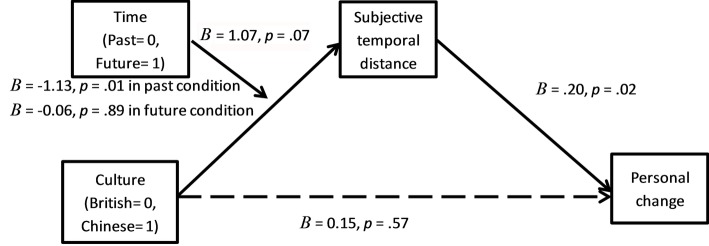
Time condition moderated the effect of culture on subjective temporal distance, which positively predicted personal change.

We predicted that Chinese would perceive the past selves as being closer to the present than would British, which would make the asymmetry in recalled and anticipated personal changes disappear for Chinese. In order to further examine this prediction, a bootstrapping analysis with culture as the predictor, time condition as the moderator, subjective temporal distance as the mediator, and personal change as the outcome was conducted. As in the previous analysis, the culture by time interaction was marginally significant, *B* = 1.07, *t* = 1.82, *p* = 0.07, and the 95% CI was (−0.10, 2.24). British perceived their past selves as being further away than did Chinese participants, *B* = −1.13, *p* = 0.01, 95% CI was (−1.99, −0.26) whereas British and Chinese perceived their future selves as equally distant, *B* = −0.06, *p* = 0.89. The effect of subjective temporal distance on personal change was significant, *B* = 0.20, *t* = 2.33, *p* = 0.02, and the 95% CI was (0.03, 0.37). The further away participants felt their selves were from the present, the more personal change they reported. The direct effect of culture on personal change was not significant, *p* = 0.57. The conditional indirect effect of culture on personal change was significant in the past condition, *B* = −0.22, and the 95% CI was (−0.66, −0.01), and nonsignificant in the future condition, *B* = −0.01, and the 95% CI was (−0.20, 0.16). Thus, subjective temporal distance accounted for the cultural asymmetry between recalled and anticipated personal changes. See Figure 7.

## General Discussion

In four studies, we found that for British participants, anticipated changes in personality (Study 1), basic values (Study 2a), and the person as a whole (Studies 2b and 3) over a period of time in the future were smaller than the recalled changes over the same length of time in the past, whereas the recalled and anticipated changes were similar for Chinese. This cross-cultural difference holds true not only for changes derived from the past, the present, and the future personal attributes but also for directly reported changes. In addition, we found that both British and Chinese participants held the lay belief that things tend to change more over a long period time than over a short period of time. Furthermore, subjective temporal distance accounted for the cross-cultural variation in recalled and anticipated changes. British participants perceived their past selves as being further away than did Chinese, and subjective temporal distance was used as a cue to infer change. Thus, this research is not only the first one to demonstrate a cultural asymmetry between recalled and anticipated changes, but also provides an explanation for the asymmetry.

Given that people use subjective temporal distance as a cue to infer change, and that Westerners anticipated that they would change less in the future than they had changed in the past, one would speculate that Westerners may perceive future times as being closer than the equidistant past ones. Indeed, Study 2b provided support for this. British participants perceived their future selves in 10 years as being closer to the present than their past ones 10 years ago at a marginal significant level. Other research has also confirmed this. Caruso et al. (2013) found that American participants perceived future times and holidays as being closer to them than past ones.

The research has significant theoretical implications. With participants at various ages, Quoidbach et al. (2013) found that anticipated changes in the next 10 years by Westerners at one age were smaller in magnitude than recalled changes in the past 10 years by other Westerners who were 10 years older. They argued that it was at least partially due to the underestimation of future changes, which could lead to decisions that the future selves would regret. However, although the methods and specific comparisons were different, the current findings suggest that both the recalled and anticipated changes were at least partially inferred from perceived temporal distance. Thus, the asymmetry is possibly a result of an overestimation of past change for Western participants, although we were not able to directly examine it due to the cross-sectional data.

Although Quoidbach et al. (2013)’s and the present research were both about the asymmetry between recalled and anticipated changes, the phenomena investigated were different. Quoidbach and colleagues compared anticipated changes by participants at one age with recalled changes by participants who were older, and the asymmetry was considered mainly as a result of the underestimation of future changes. They considered self-enhancement motivation as one possible reason for the asymmetry, i.e., people believe that they had improved a lot in the past, and achieved a high level in their traits and values at the present, leaving little room for further improvement. In the current research, we investigated how people perceive the magnitudes of past and future changes differently. Anticipated and recalled changes from participants of the same age were compared. Our data suggest that self-enhancement motivation cannot explain the cultural variations in the asymmetry in recalled/anticipated changes. Instead, such cultural variations were accounted for by subjective temporal distance, suggesting that people’s lay theories regarding the association of time and change influence people’s estimation of personal change.

Although the present paper focused on personal changes, the association between subjective temporal distance and perceived change implies that similar findings would be obtained in other domains, such as perceived changes in other people, events, relationships, etc. This is because most things would change with time. It is thus likely that people would also use their subjective temporal distance as a cue to infer the amount of change in other domains. We are currently investigating this prediction to generalize the results across contexts.

The finding that subjective temporal distance accounted for the cultural variation in recalled and anticipated changes implies a way to reduce misconceptions about our past and future selves. We are currently investigating whether the misconception could be reduced, e.g., by reminding ourselves about how long the temporal distance actually is. Alternatively, one can try to think about personal milestones (such as birthdays, graduation, etc.) and transitions (such as changing jobs, moving to different cities, etc.) that happened in the past or might happen in the future, which could make him or her feel that the past or future is subjectively further away from the present (Wilson and Ross, 2003), and thus could possibly reduce the misconception in the desired way.

The current paper has several limitations. Previous research has shown that GDP positively correlated with looking forward into the future (Preis et al., 2012). Given that we had data from two countries/regions only, we were not able to include GDP data as a control variable. Future studies can investigate how GDP and income data may have influence on the results. In addition, big five personality traits and basic values were used in Studies 1 and 2a. Some researchers found that the big five personality factor did not accurately describe Chinese personalities (Wang et al., 2005). However, this potential cultural bias in measurement could not fully account for the findings. First, a large number of other studies showed that the big five personality traits and basic values were replicable across cultures, including China (McCrae and Costa, 1997; McCrae and Terracciano, 2005; Terracciano and McCrae, 2006; Schwartz, 2012). Thus, the personality and basic value measurements in these studies could be appropriate for Chinese participants as well. Furthermore, additional analysis revealed that absolute value changes derived from the basic value measurement positively correlated with directly reported value changes for Chinese in Study 2a, *r* = 0.31, *p* < 0.01, suggesting that the basic value measurement was valid for Chinese. In addition, the personality and value changes for Chinese participants in both the past and future were not smaller in magnitude than recalled personality and value changes for British participants in the future, suggesting that Chinese did not have lower sensitivity to the measurements in general. Furthermore, the findings were conceptually replicated with directly reported personal changes in Studies 2b and 3. Thus, it is unlikely that the potential insensitivity for Chinese participants accounted for all the findings. That being said, it is still worth replicating the studies with other culturally developed personality measures in future studies.

## Ethics Statement

The research protocol was approved by the Ethical Review Committee at University of Macau, approval No. MYRG2016-00220-FSS. Participants received a letter of information which outlines the research project, and then signed a consent form or chose “agree to participate” option before they participated in the studies.

## Author Contributions

TG developed the study concept, analyzed the data, and drafted the manuscript. RS provided critical revisions. Both authors contributed to the study design and data collection and approved the final version of the manuscript for submission.

### Conflict of Interest Statement

The authors declare that the research was conducted in the absence of any commercial or financial relationships that could be construed as a potential conflict of interest.
